# The Representation of Gender and Race/Ethnic Groups in Randomized Clinical Trials of Individuals with Systemic Lupus Erythematosus

**DOI:** 10.1007/s11926-018-0728-2

**Published:** 2018-03-17

**Authors:** Titilola Falasinnu, Yashaar Chaichian, Michelle B. Bass, Julia F. Simard

**Affiliations:** 1 Department of Health Research and Policy, Stanford School of Medicine, 150 Governor’s Lane, Stanford, CA 94305 USA; 2 Department of Medicine, Division of Immunology and Rheumatology, Stanford School of Medicine, 900 Blake Wilbur Dr, 2nd Fl, Stanford, CA 94305 USA; 3 Lane Medical Library and Knowledge Management Center, Stanford University Medical Center, 300 Pasteur Drive, L109, Stanford, CA 94305 USA; 4 Division of Immunology and Rheumatology, Department of Health Research and Policy, Stanford School of Medicine, 150 Governor’s Lane, Stanford, CA 94305 USA

**Keywords:** Clinical trials, Race, Ethnicity, Representation, Enrollment

## Abstract

**Purpose of Review:**

This review evaluated gender and race/ethnic representation in randomized controlled trials (RCTs) of patients with systemic lupus erythematosus (SLE).

**Recent Findings:**

Whites comprise 33% of prevalent SLE cases and comprised 51% of RCT enrollees. Blacks encompass 43% of prevalent SLE cases, but only represented 14% of RCT enrollees. Hispanics comprise 16% of prevalent SLE cases and 21% of RCT enrollees, while Asians comprise 13% of prevalent SLE cases and 10% of RCT enrollees. Males encompass 9% of SLE cases and 7% of RCT enrollees. The reporting and representation of males have remained stable over time, although their representation in RCTs is slighter lower than the prevalence of SLE in males. The representation of Hispanics, Asians, and Native Americans increased over time. However, the representation of blacks among RCT participants has decreased since 2006–2011.

**Summary:**

RCTs among SLE patients need larger sample sizes in order to evaluate heterogeneity in outcomes among racial subgroups. It is imperative that novel strategies be developed to recruit racial minorities with SLE by identifying and improving barriers to RCT enrollment in order to better understand the disease’s diverse population.

## Introduction

The most salient aspect of the epidemiology of systemic lupus erythematous (SLE) is the preponderance of females and racial/ethnic minorities among cases. Sex hormones, environmental triggers, and genetic factors have been implicated in the etiological pathway for these disparities [1]. The average prevalence ratio of females to males has been reported to be 9:1 across multiple populations [2]. Although males comprise the minority of SLE cases and are often described as having lower disease risk, studies indicate that males tend to present with more severe sequelae compared to females [3]. For example, males are more likely to present with higher SLE disease activity and organ damage [4•], more frequent manifestations of seizures and neuropsychiatric conditions [5, 6], and faster progression to fulminant renal disease compared to women [7].

The race-related heterogeneity in disease presentation and susceptibility often indicates that racial minorities are more likely to incur an excess burden in disease risk and comorbidities compared to whites [8••]. For example, in a recent study of Medicaid enrollees in the United States (US), blacks were twice as likely to be living with SLE compared to whites [9]. Asians and Hispanics have lower prevalence and incidence compared to blacks; however, these racial groups are more likely to present with severe manifestations of the disease compared to whites [9, 10]. A few studies have explored what is termed the Hispanic and Asian paradox, where these groups have lower mortality compared to whites, blacks, and Native Americans [11••] and lower risk of cardviovascular disease compared to whites and blacks [12].

Much of the discourse on these gender and racial/ethnic disparities has focused on incidence/prevalence, disease severity, and mortality. In randomized controlled trials (RCTs) of more common chronic diseases such as cardiovascular disease, cancer, and diabetes, racial/ethnic disparities have been reported; specifically, racial minorities are vastly under-represented in comparison with whites and the representation of blacks has decreased over time [13–16]. In 1993, the NIH Revitalization Act was signed into law in part to promote the inclusion of racial minorities in RCTs [16]. Despite recognition that SLE disease susceptibility and severity differ by race/ethnicity and gender, the full scope of how adequately racial minorities and males are included within SLE RCTs has yet to be characterized. Little is also known about the accuracy of race and ethnicity classification in SLE RCTs, for example, whether race and ethnicity are reported as separate categories, are included together, or are even mentioned.

It is important to ensure that those with the highest disease burden and those with severe disease manifestations are adequately represented in RCTs that may produce therapeutics impacting their disease trajectory. We conducted a scoping review [17] to determine the prevalence of race/ethnic groups and males in SLE RCTs and examined whether their inclusion has changed over time. We also determined factors associated with racial/ethnic and male enrollment.

## Methods

**Fig. 1:**
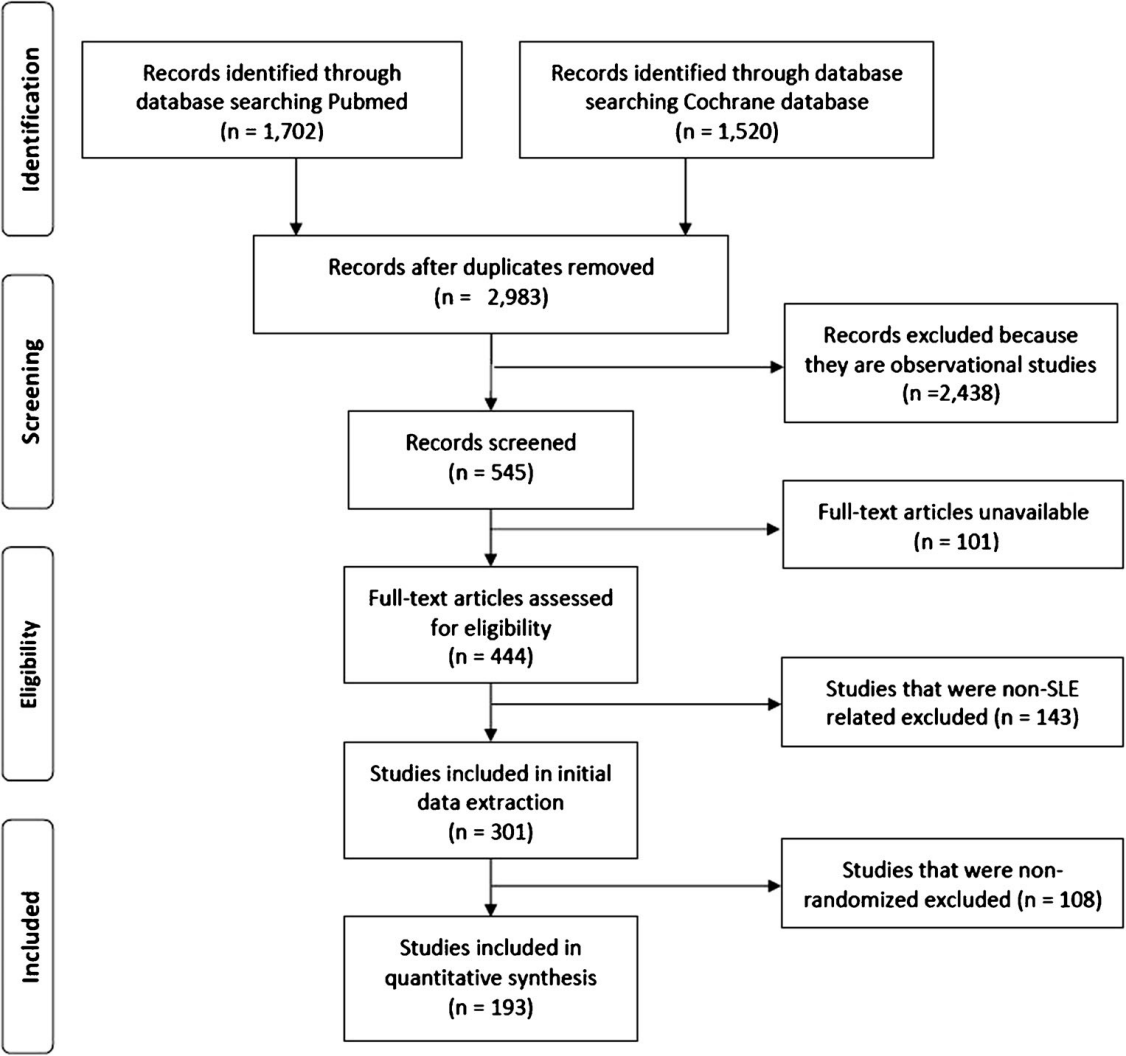
The attrition diagram for literature search. After eliminating observational studies (*n* = 2438), unavailable full text articles (*n* = 101), studies that were not SLE-related (*n* = 143) and non-randomized studies (*n* = 108), 193 studies were retained for this review

The medical librarian developed the search strategies in consultation with the first author for the concepts of systemic lupus erythematosus or lupus nephritis. We conducted a PubMed (1947-)and Cochrane Library of Systematic Reviews (2003-) search limiting the time period to January 1997 to July 2017 to identify randomized clinical trials (RCTs) conducted among SLE patients. The search spanned this 20-year period in order to arrive at a manageable number of articles. Please see appendix for complete search strategies. Search criteria were defined to include the following keywords: “systemic lupus erythematosus” and “lupus nephritis” in the title, abstract, or body of the articles. We excluded studies that did not include randomization or included patients with other types of lupus that were not SLE, e.g., cutaneous-only lupus or drug-induced lupus. Animal studies were also excluded. Studies for inclusion were also limited by English language only. A total of 2983 unique records were identified from the two databases after duplicates were removed using the EndNote software deduplication feature. After eliminating observational studies (*n* = 2438), unavailable full text articles (*n* = 101), studies that were not SLE-related (*n* = 143) and non-randomized studies (*n* = 108), 193 studies were retained for this review (Fig. 1).

### Data Extraction

After removing duplicates from the initial search and abstract screening, the full text of the remaining 193 articles were reviewed. Study data were collected and managed using Google Forms, an electronic data capture tool hosted through Google. We extracted the following domains from each article: geographical location, participant age category (i.e., pediatric, adult, or both), inclusion and participant counts by gender and by racial/ethnic groups, type of intervention under study (i.e., drug or other), names and class of drug therapies in interventions, year of publication, total number of participants, and funding type (NIH, industry, both or not stated). For the purpose of this review, we approximated the race and ethnic categorizations defined by the United States Census Bureau’s guidelines [18] to fit with reporting trends in the literature: American Indian or Alaskan Native; Asian or Native Hawaiian or Other Pacific Islander; black or African American; and white. Hispanic ethnicity was documented as a race group in some papers and as an ethnicity in others. To reconcile this inconsistency, we documented all participants who were recorded as either Hispanic ethnicity or Hispanic race as “Hispanic.” Thus, the following were race/ethnic categories examined in this review: white, black, Hispanic, Asian, and Native American.

### Examination of the Reporting of Male Representation

First, we examined the proportion of RCTs reporting the inclusion of male participants. We defined studies that included both male and female participants as those who had male representation (note: no studies solely included male participants). We examined whether the inclusion of male participants varied by trial characteristics, e.g., geographical location, participant age category, type of intervention under study, class of drug, funding type, and publication year. We calculated the overall proportion of males included in the study by dividing the sum of males in all the RCTs by the total recruitment. We also compared the proportion of males in RCTs to the proportion of males in published prevalence studies of SLE patients [9, 10, 19, 20] and examined whether the proportion of males included in RCTs varied over time.

### Examination of the Reporting and Representation of Race/Ethnicity

Second, for an examination of the *reporting* of race/ethnic distribution of participants in studies, we focused on studies that included participants in the US. We examined whether studies that reported the race/ethnic composition of their participants varied by RCT characteristics. We calculated the proportion of studies that reported the presence of each race/ethnic group and the characteristics that were associated with the inclusion of these groups (Table 2).

We defined race/ethnic *representation* as the proportion of total enrolles in all the RCTs that belonged to each race/ethnic group. We compared the proportion of race/ethnic groups in RCTs to the proportion of race/ethnic groups in published prevalence studies of SLE patients. We also examined whether the proportion of race/ethnic groups included varied over time.

## Results

### Baseline Characteristics of Included RCTs

Most studies (69%) enrolled both male and female participants and 26% of the studies had only female enrollees (Table 1). Forty-one percent of the studies included US sites. Eleven percent of studies reported race and ethnicity as separate categories in accordance with the US Census Bureau’s guidelines for reporting race and ethnicity, and 37% reported neither. A majority of studies (52%) did not report the funding source, 37% were industry-funded, and a minority were NIH-funded (6%) or jointly funded by the NIH and industry sources (5%). Most RCTs in this review were composed of 50 enrollees or less (43%) while 19% of the studies included 200 or more enrollees. The most commonly investigated drugs were biologic disease-modifying anti-rheumatic drugs (DMARDs) (23%), followed by conventional DMARDs (20%) and alkylating agents (predominantly cyclophosphamide) (20%). Approximately 85% of studies enrolled only adults and 5% comprised only pediatric patients. A minority (30%) of studies enrolled patients with lupus nephritis. The largest proportion of studies (43%) were published recently, between 2012 and 2017.

**Table 1:** Characteristics of the 193 included studies

	No. of trials (%)	% Enrolling male^a^
Overall	193 (100.0)	134 (69.4%)
Gender distribution		
Both males and females	134 (69.4)	134 (100.0%)
Females only	50 (25.9)	N/A
Gender not reported	9 (4.7)	
Location		
US included	79 (40.9)	62 (78.5)
US not included	101 (52.3)	64 (63.4)
Unknown	13 (6.7)	8 (61.5)
Race/ethnicity reporting		
Ethnicity only reported	5 (2.6)	3 (60.0)
Hispanic reported as a separate race group	28 (14.5)	23 (82.1)
Race only reported (and Hispanic ethnicity was not mentioned)	67 (34.7)	48 (71.6)
Race and ethnicity reported as separate categories	22 (11.4)	21 (95.5)
Neither race nor ethnicity was reported	71 (36.8)	39 (54.9)
Funding source		
Industry	72 (37.3)	58 (80.6)
NIH	12 (6.2)	9 (75.0)
Jointly funded	9 (4.7)	6 (66.7)
Not stated	100 (51.8)	61 (61.0)
Total enrolment, no. of patients		
≤ 50	84 (43.5)	51 (60.7)
51 to 100	47 (24.4)	35 (74.5)
101 to 200	26 (13.5)	16 (61.5)
≥ 200	36 (18.7)	32 (88.9)
Intervention		
Drug	174 (90.2)	127 (73.0)
Other	19 (9.8)	7 (36.8)
Drugs under study^b^		
Alkylating agent	39 (20.2)	33 (84.6)
Biologic DMARD	45 (23.3)	44 (97.8)
Conventional DMARD	39 (20.2)	36 (92.3)
Glucocorticoids	22 (11.4)	18 (81.8)
Hormonal therapy	27 (14.0)	3 (11.1)
Other	63 (32.6)	37 (58.7)
Population		
Adults	163 (84.5)	112 (68.7)
Pediatrics	10 (5.2)	6 (60.0)
Both	13 (6.7)	12 (92.3)
Unknown	7 (3.6)	4 (57.1)
Study included patients with lupus nephritis		
Yes	58 (30.1)	52 (89.7)
No	135 (69.9)	82 (60.7)
Publication year		
1997–2001	14 (7.3)	11 (78.6)
2002–2006	38 (19.7)	18 (47.4)
2007–2011	58 (30.1)	43 (74.1)
2012–2017	83 (43.0)	62 (74.7)

^a^This column represents the proportion of each row that included male representation. For example, 64 (63.4%) out of 101 US-based studies included male enrollees

^b^This category does not total 100% because some RCTs may evaluate more than one of these therapeutic ages

### Study Characteristics Associated with the Inclusion of Males

We determined study characteristics that were associated with including males (Table 1) (*n* = 134). We found that studies reporting race and ethnicity categories in accordance with the United States Census Bureau were more likely to also enroll male patients (96%) compared to those that reported neither race nor ethnicity (55%). Industry-funded studies were more likely (81%) to have male representation compared to those funded by the NIH (67%) or those that were jointly funded by industry (75%). Studies with large sample sizes (i.e., 200 enrollees or more) were more likely to have male representation than those with small sample sizes (i.e., 50 enrollees or less), 89 vs 61%, respectively. Studies investigating biologic DMARDs (98%) had more male representation than those investigating conventional DMARDs (92%), alkylating agents (85%), glucocorticoids alone (82%), and other drugs (59%). Studies that included both adult and pediatric patients were most likely to enroll male participants (92%) in comparison with those enrolling adults only (69%) or pediatric patients only (60%). Studies that enrolled patients with lupus nephritis were more likely to include male representation compared to those that did not enroll patients with lupus nephritis (90 vs 61%, respectively). Male representation did not appear to vary over time.

### Characteristics of US Study Sites

We limited our study sample to studies that had at least one US enrollment site (*n* = 79) to evaluate race/ethnic representation in the USA (Table 2). A majority of these studies (79%) included both male and female participants. One-quarter of the studies reported race and ethnicity as separate categories, while 9% of studies reported neither. Approximately 49% of these studies were industry-funded, 15% were NIH-funded, 11% were funded by both industry and NIH, and 24% did not state the funding source. Thirty-eight percent of studies comprised 200 or more participants, while 32% had 50 or fewer participants. Biologic DMARDs were the most commonly investigated drugs (42%), while alkylating agents (14%) and hormonal therapies (14%) were the second most frequent. Lupus nephritis was investigated in 27% of the RCTs. The largest proportion of studies (48%) were published between 2012 and 2017.

**Table 2:** Characteristics of the 79 studies in the USA

	Total no. of trials (%)	% of RCTs that reported race/ethnic composition	% of RCTs that reported at least one participant that was…				
			White	Black	Hispanic	Asian	Native American
Overall	79	72 (91.1)	68 (86.1)	64 (81.0)	47 (59.5)	50 (63.3)	25 (31.7)
Sex distribution							
Both males and females	62 (78.5)	59 (95.2)	57 (91.9)	53 (85.5)	41 (66.1)	42 (67.7)	22 (35.5)
Females only	15 (19.0)	12 (80.0)	10 (66.7)	10 (66.7)	6 (40.0)	7 (46.7)	2 (13.3)
Sex not reported	2 (2.5)	1 (50.0)	1 (50.0)	1 (50.0)	0 (0.0)	1 (50.0)	1 (50.0)
Race/ethnicity reporting							
Ethnicity only reported	2 (2.5)	2 (100.0)	1 (50.0)	1 (50.0)	1 (50.0)	1 (50.0)	0 (0.0)
Race only reported (and Hispanic ethnicity was not mentioned)	25 (31.6)	25 (100.0)	23 (92.0)	20 (80.0)	1 (4.0)	12 (48.0)	4 (16.0)
Hispanic reported as a separate race group	25 (31.6)	25 (100.0)	25 (100.0)	24 (96.0)	25 (100.0)	21 (84.0)	11 (44.0)
Race and ethnicity reported as separate categories	20 (25.3)	20 (100.0)	19 (95.0)	19 (95.0)	20 (100.0)	16 (80.0)	10 (50.0)
Neither race nor ethnicity was reported	7 (8.9)	0 (0.0)	0 (0.0)	0 (0.0)	0 (0.0)	0 (0.0)	0 (0.0)
Funding source							
Industry	39 (49.4)	38 (97.4)	37 (94.9)	35 (89.7)	24 (61.5)	28 (71.8)	13 (33.3)
NIH	12 (15.2)	12 (100.0)	10 (83.3)	11 (91.7)	5 (41.7)	7 (58.3)	5 (41.7)
Jointly funded	9 (11.4)	8 (88.9)	7 (77.8)	6 (66.7)	8 (88.9)	5 (55.6)	3 (33.3)
Not stated	19 (24.1)	14 (73.7)	14 (73.7)	12 (63.2)	10 (52.6)	10 (52.6)	4 (21.1)
Total enrolment, no. of patients							
≤ 50	25 (31.6)	21 (84.0)	20 (80.0)	18 (72.0)	14 (56.0)	14 (56.0)	10 (40.0)
51 to 100	12 (15.2)	11 (91.7)	9 (75.0)	11 (91.7)	6 (50.0)	7 (58.3)	1 (8.3)
101 to 200	12 (15.2)	11 (91.7)	11 (91.7)	10 (83.3)	5 (41.7)	8 (66.7)	3 (25.0)
≥ 200	30 (38.0)	29 (96.7)	28 (93.3)	25 (83.3)	22 (73.3)	21 (70.0)	11 (36.7)
Intervention							
Drug	73 (92.4)	67 (91.8)	64 (87.7)	60 (82.2)	45 (61.6)	48 (65.8)	24 (32.9)
Other	6 (7.6)	5 (83.3)	4 (66.7)	4 (66.7)	2 (33.3)	2 (33.3)	1 (16.7)
Drugs under study^a^							
Alkylating agent	11 (13.9)	9 (81.8)	9 (81.8)	8 (72.7)	8 (72.7)	7 (63.6)	3 (27.3)
Biologic DMARD	33 (41.8)	31 (93.9)	30 (90.9)	29 (87.9)	20 (60.6)	25 (75.8)	14 (42.4)
Conventional DMARD	9 (11.4)	8 (88.9)	8 (88.9)	8 (88.9)	8 (88.9)	7 (77.8)	2 (22.2)
Glucocorticoids	6 (7.6)	4 (66.7)	4 (66.7)	4 (66.7)	4 (66.7)	4 (66.7)	4 (66.7)
Hormonal therapy	11 (13.9)	10 (90.9)	10 (90.9)	8 (73.7)	5 (45.5)	6 (54.6)	2 (18.2)
Other	17 (21.5)	15 (88.2)	13 (76.5)	13 (76.5)	10 (58.8)	8 (47.1)	3 (17.6)
Population							
Adults	65 (82.3)	59 (90.8)	56 (86.2)	53 (81.5)	35 (53.8)	40 (61.5)	20 (30.8)
Pediatrics	6 (7.6)	6 (100.0)	5 (83.3)	4 (66.7)	5 (83.3)	3 (50.0)	1 (16.7)
Both	6 (7.6)	6 (100.0)	6 (100.0)	6 (100.0)	6 (100.0)	6 (100.0)	3 (50.0)
Unknown	2 (2.5)	1 (50.0)	1 (50.0)	1 (50.0)	1 (50.0)	1 (50.0)	1 (50.0)
Study included patients with lupus nephritis							
Yes	21 (26.6)	18 (85.7)	18 (85.7)	16 (76.2)	15 (71.4)	15 (71.4)	5 (23.8)
No	58 (73.4)	54 (93.1)	50 (86.2)	48 (82.8)	32 (55.2)	35 (60.3)	20 (34.5)
Publication year							
1997–2001	6 (7.6)	4 (66.7)	4 (66.7)	3 (50.0)	3 (50.0)	3 (50.0)	1 (16.7)
2002–2006	15 (19.0)	12 (80.0)	12 (80.0)	10 (66.7)	8 (53.3)	8 (53.3)	3 (20.0)
2007–2011	20 (25.3)	20 (100.0)	20 (100.0)	18 (90.0)	14 (70.0)	14 (70.0)	7 (35.0)
2012–2017	38 (48.1)	36 (94.7)	32 (84.2)	33 (86.8)	22 (57.9)	25 (65.8)	14 (36.8)

^a^This category does not total 100% because some RCTs may evaluate more than one of these therapeutic ages

### Characteristics of US Study Sites Reporting Race Composition

Studies including both male and female enrollees were more likely (95%) to report race composition compared with those with females only (80%) and those that did not report gender composition (50%) (Table 2). All NIH-only funded studies reported race composition, compared with 97% of industry-funded and 89% of jointly funded studies. Studies with large sample sizes (i.e., 200 enrollees or more) were more likely to report race composition than those with small sample sizes (i.e., 50 enrollees or less), 97 vs 84%, respectively. Studies investigating biologic DMARDs (94%) and studies involving glucocorticoids were most likely to report race composition (67%). All studies composed of only pediatric patients reported race composition, in comparison to 91% of studies including only adults. Studies that evaluated lupus nephritis were less likely than those that did not report race composition (86 vs 93%, respectively). There appeared to be an increase in the reporting of race composition from 1997 to 2001 (67%) until 2007–2011 (100%).

### Characteristics of US Study Sites Reporting Specific Racial/Ethnic Group Representation

We also examined the representation of specific race and ethnic groups among RCTs in the USA (Table 2). Here, we focus on the representation of blacks, Hispanics, Asians, and Native Americans. Sixty-four (81%) of US-based studies reported having black participants. The following characteristics were associated with reporting the inclusion of black enrollees: inclusion of both adult and pediatric patients (100%), NIH-funding (92%), enrolling 51 to 100 participants (92%), studies published between years 2007 and 2011 (90%), investigating conventional DMARDs (89%), and studies that did not include patients with lupus nephritis (83%). Forty-seven (60%) of US-based studies reported Hispanic participants. The following characteristics were associated with reporting Hispanic enrollees: inclusion of both adult and pediatric patients (100%), joint funding by the NIH and industry (89%), investigating conventional DMARDs (89%), enrolling 200 or more participants (73%), studies that did not include patients with lupus nephritis (71%), studies that did not include patients with lupus nephritis (70%), and inclusion of both male and female enrollees (66%).

Fifty (63%) of US-based studies reported having Asian participants. The same study characteristics were associated with including Asian as compared to Hispanic enrollees, with the exception of industry-only funding compared to joint funding by the NIH and industry. Twenty-five (32%) of the US-based studies reported having Native American participants and few characteristics were associated with including Native American participants (Table 2).

### Temporal Trends in Race/Ethnicity Reporting and Representation

**Fig. 2:**
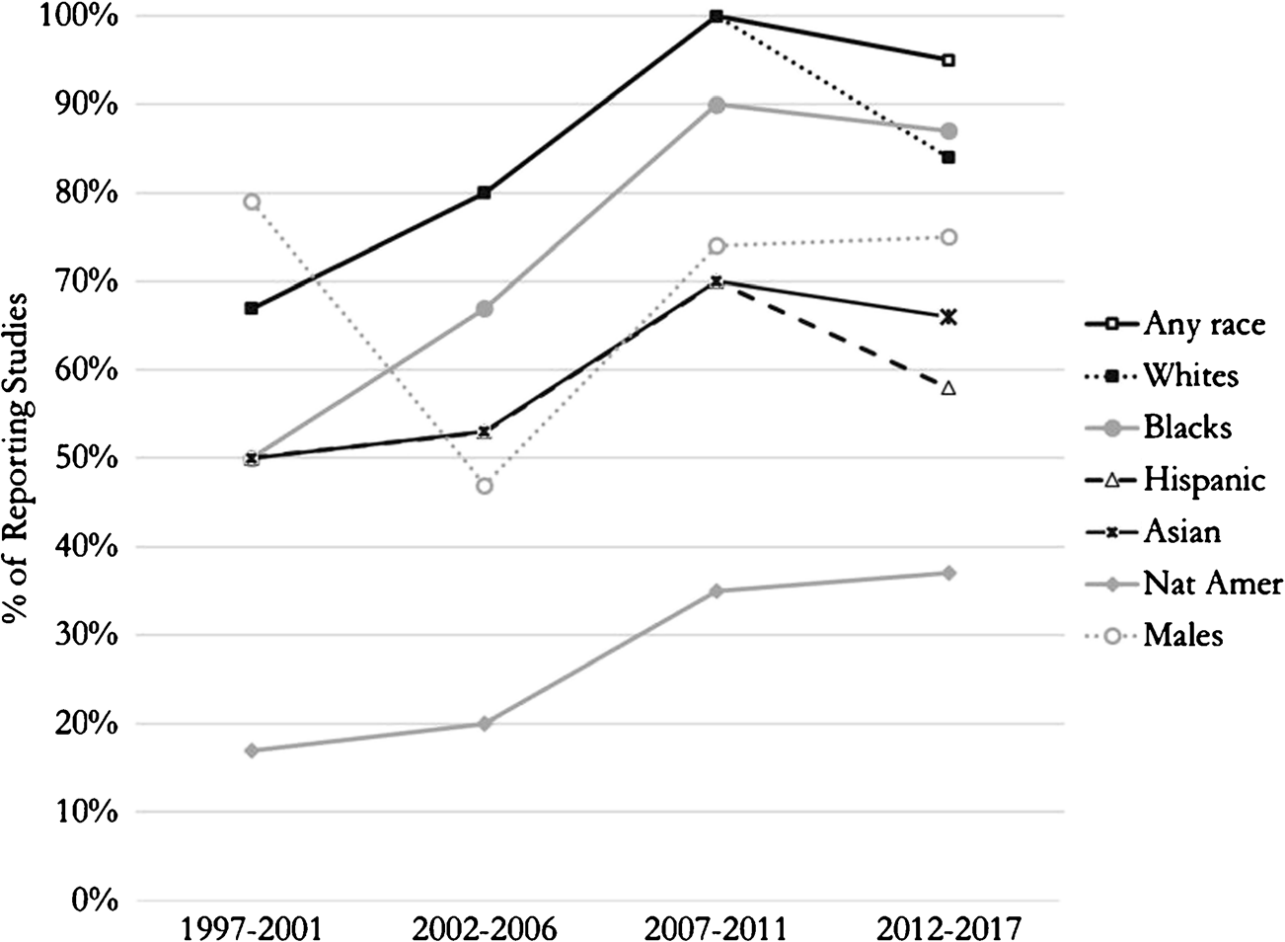
Race and gender reporting over time. Percentages of studies reporting representation of males, whites, blacks, Hispanics, or Asians from 1997 to 2017. Reporting of most race/ethnic groups appears to peak in the period 2007–2011, after which there appears to be a decrease. However, the reporting of Native American inclusion increased over time

**Fig. 3:**
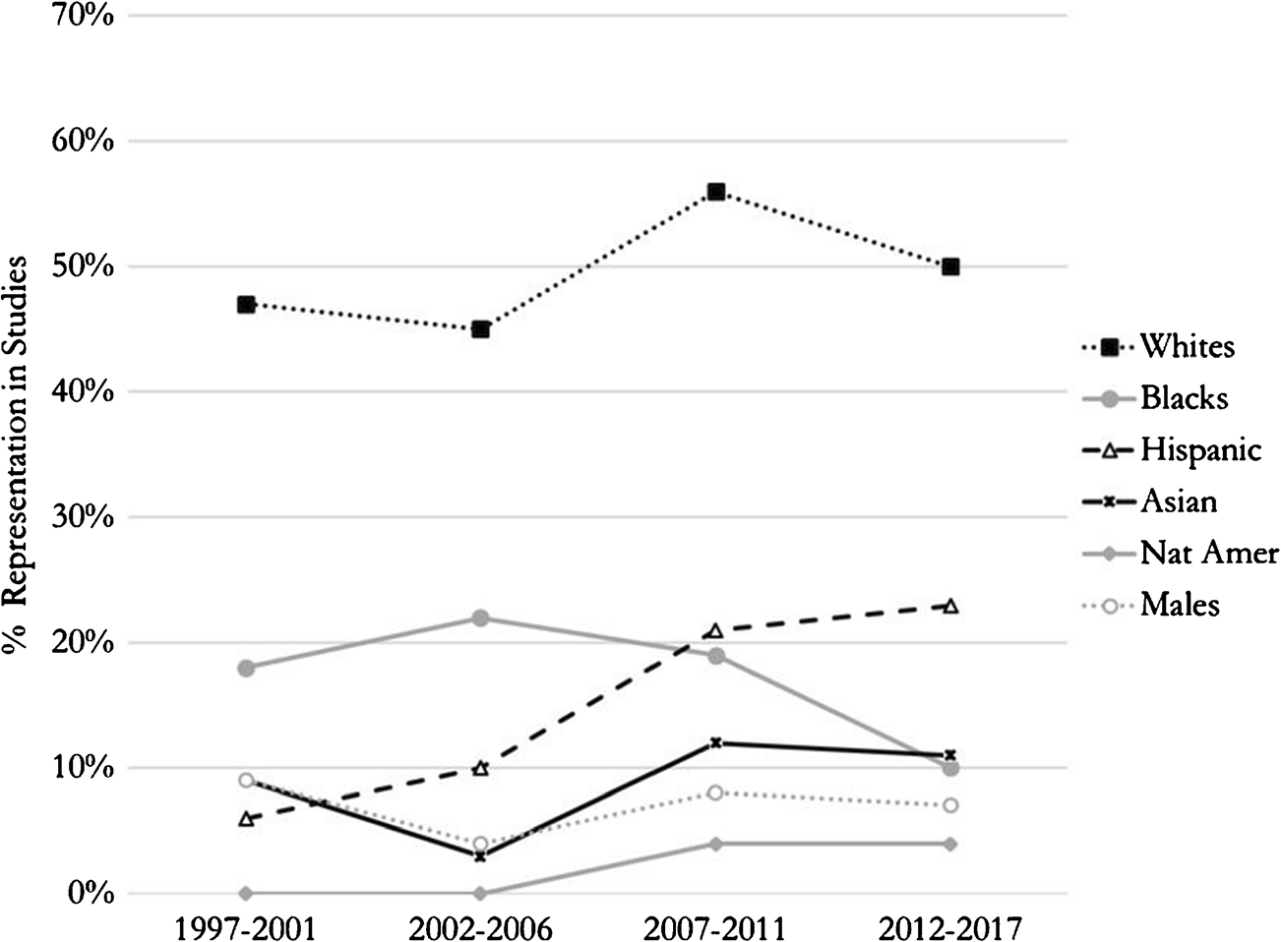
Race and gender representation over time. Average percentages of race and male enrollments in race-reporting trials from 1997 to 2017. Whites represented between 47 and 56% of RCT enrollees between the periods 1997–2001 and 2012–2017; however, blacks comprised 18 and 10% of enrollees, respectively. There appears to be an increase in the representation of racial minority groups other than blacks until the period 2007–2011, after which there appears to be a decrease. Hispanics comprised 6% of enrollees in the period 1997–2001, but this increased to 23% of enrollees in the period 2012–2017. The inclusion of Asians increased from 9% in 1997–2001 to 11% in 2012–2017. Native Americans increased from 0 to 4% during those two periods

Reporting of most race/ethnic groups appears to peak in the period 2007–2011, after which there appears to be a decrease (Fig. 2). However, the reporting of Native American inclusion increased over time. Figure 3 shows the representation of race and gender over time. Whites represented between 47 and 56% of RCT enrollees between the periods 1997–2001 and 2012–2017; however, blacks comprised 18 and 10% of enrollees, respectively. There appears to be an increase in the representation of racial minority groups other than blacks until the period 2007–2011, after which there appears to be a decrease. Hispanics comprised 6% of enrollees in the period 1997–2001, but this increased to 23% of enrollees in the period 2012–2017. The inclusion of Asians increased from 9% in 1997–2001 to 11% in 2012–2017. Native Americans increased from 0 to 4% during those two periods.

### Race/Ethnicity Representation in SLE RCTs Compared with SLE Prevalence Studies

**Fig. 4:**
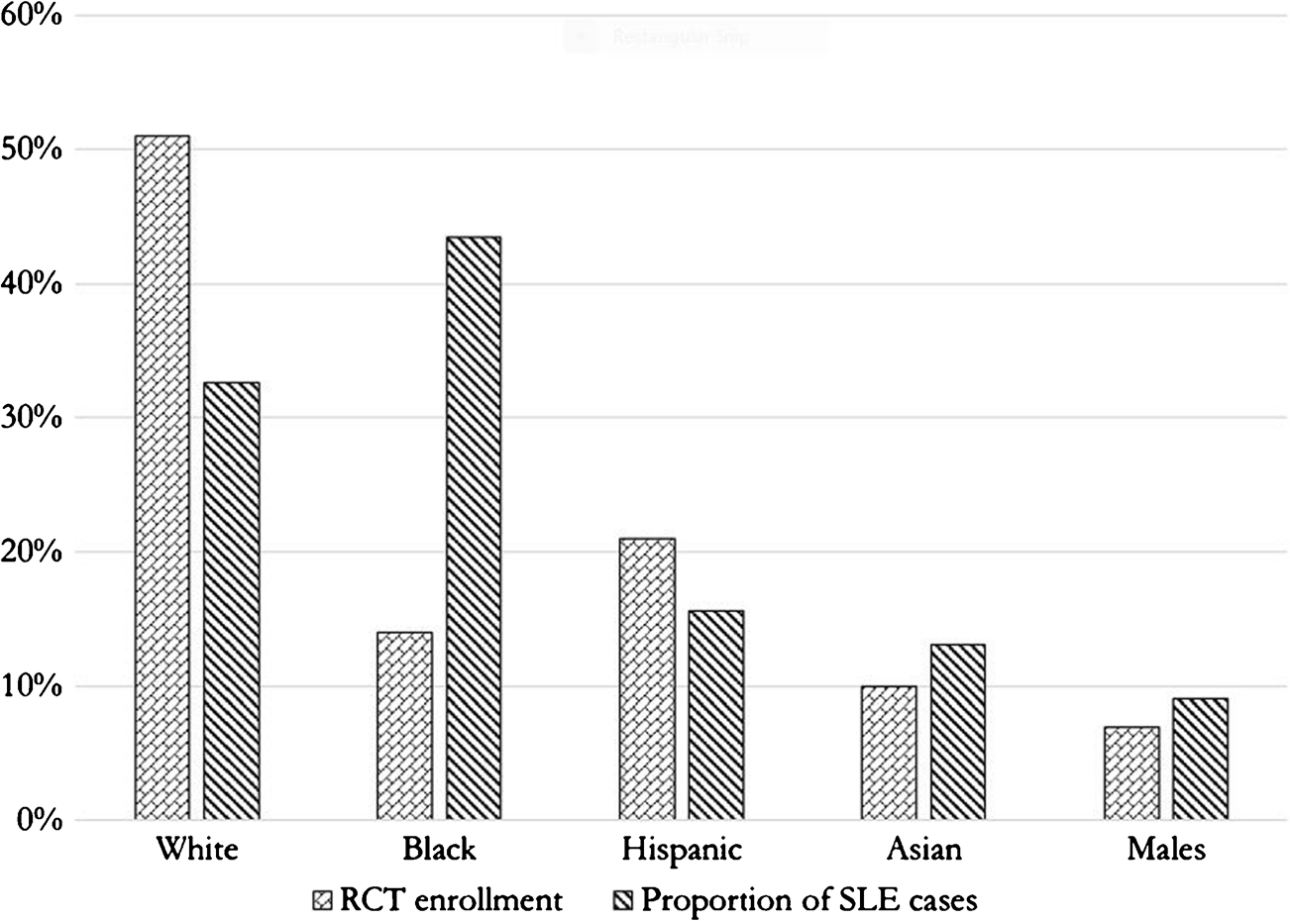
Compares the overall race and gender representation with recently published prevalence estimates. While whites comprised 33% of prevalent SLE cases in the USA [9, 10, 19, 20], they comprised 51% of RCT enrollees. Blacks comprised 43% of prevalent SLE cases [9, 10, 19, 20], but only 14% of RCT enrollees. Hispanics encompassed 16% of prevalent SLE cases [9, 10, 19, 20] and 21% of RCT enrollees, while Asians comprised 13% of prevalent SLE cases and 10% of RCT enrollees

Figure 4 compares the overall race and gender representation with recently published prevalence estimates. While whites comprised 33% of prevalent SLE cases in the USA [9, 10, 19, 20], they comprised 51% of RCT enrollees. Blacks comprised 43% of prevalent SLE cases [9, 10, 19, 20], but only 14% of RCT enrollees. Hispanics encompassed 16% of prevalent SLE cases [9, 10, 19, 20] and 21% of RCT enrollees, while Asians comprised 13% of prevalent SLE cases and 10% of RCT enrollees. We were unable to calculate prevalence estimates of Native Americans from the literature. Males encompassed 9% of prevalent SLE cases and 7% of RCT enrollees.

## Discussion

Although the representation of males has been stable over time, their representation in RCTs is slighter lower than the male prevalence of SLE. Males were included in 69% of RCTs in this review (25% of studies were comprised solely of female participants, where the authors examined female-related issues such as hormone replacement therapy and birth control, and 5% of studies did not report the gender composition of their participants). Removing these female-only studies from the denominator, male representativeness only increased from 7 to 8%. While the focus on fertility and reproduction issues is of great interest in females because SLE disproportionately impacts women of child-bearing age, there is also a need to understand the role that hormones other than “female hormones” play in the pathophysiology of the disease—only three studies that included males evaluated the roles of these other hormones. Our findings emphasize the need for increased enrollment of males in SLE RCT as this under-representation may contribute to diminutive understanding of the disease in males, especially on matters related to deleterious effects of drug therapies (and the natural course of this disease) .

The inclusion of Hispanics, Asians, and Native Americans increased over time, a finding that may have to do with the inclusion of RCT sites in Asian and Latin American countries, in addition to enrollment in US sites. Due to the inconsistency of the reporting of study sites, we were not able to disentangle RCTs with only US locations and examined studies that included at least one US site. We found that the representation of blacks among RCT participants has decreased since 2006–2011 in the USA, which is consistent with reviews of race/ethnic representation in other disease states [16]. Reasons for minority under-representation in RCTs are multifactorial and often involve system-level actors—e.g, clinicians, hospitals, and the community [21–24]. Historical acts of discrimination and marginalization in healthcare settings (e.g., the Tuskegee Syphilis Study) have been hypothesized as reasons why racial minorities are more distrustful of health systems and may also contribute to the under-representation of these groups in RCTs. The question of what causes racial disparities in SLE often elicits two explanations: socioeconomic factors and genetic susceptibility. First, social epidemiological inquiries into health disparities in SLE have evaluated the role that economic inequalities (i.e., income, education, geographic contexts) play in exacerbating these racial/ethnic disparities [8••]. In the USA, the impact of socioeconomic status on health outcomes cannot be overstated as it is a major determinant of who has access to health insurance and optimal access to appropriate health services. Among SLE patients, evidence suggests that race and genes are strongly predictive of disease severity at diagnosis [8••]. However, some studies indicate that the role of poverty becomes a more significant prognostic factor over the course of the disease [8••]. Poorer health literacy resulting from lower educational attainment among racial minorities has been associated with more limited access to healthcare [25].

Second, the heterogeneity in SLE presentation between whites and racial minorities is often posited to have a genetic basis. Examples of genetic predisposition to SLE among different ethnicities influencing disease susceptibility and disease expression [26, 27] include the p53 gene in Koreans that was not replicable in a white population [28]; an association of the *HSP70* gene in Africans and Spaniards that was not found among Mexicans; and the varying levels of significance of the protein receptor *CTLA4* among Asians and whites [29]. It is also widely known that an individual’s ethnic or ancestral background may determine their treatment response [30]. For example, there is a class of beta-blockers that may be less effective in a subgroup of blacks for the treatment of cardiovascular disease [30]. In the case of SLE, the traditionally employed drug for treating fulminant lupus nephritis—cyclophosphamide—has lower effectiveness for remission in black and Hispanic patients compared to whites and Asians [8••]. Moreover, there has been a recent rise in RCTs involving genotype-guided dosing, where genetic factors are taken into consideration when prescribing appropriate medication dosing [31]. While these genotype-guided dosing RCTs could inform future tailored interventions, a major concern is that the under-representation of racial minorities could lead to ineffective treatment, or even be detrimental to the disease course in these groups. Thus, the issue of representativeness is one of health equity, human rights, and social justice. In this inquiry, our main concern is why racial minorities (specifically, blacks) comprise the majority of SLE cases in the USA, but are vastly under-represented in RCTs. This leads us to introduce a possible alternative explanation for health disparities in SLE: structural and institutional factors that limit access to appropriate therapeutic options and ultimately impact treatment responses in racial minorities.

The issue of gender and race/ethnic representation is at the heart of clinical inquiry and strikes at the very notion of generalizability. External validity is the suitability of applying RCT results to other populations, either broadly or precisely [32••]. For example, the question often arises about whether the findings of an RCT are replicable in contexts that differ sociodemographically and by disease severity [32••]. Some may argue that the lack of representativeness in RCTs may be an issue of expediency where it makes economic and practical sense to only include, for instance, convenience sampling of individuals attending a clinic instead of concerning oneself about ensuring an appropriate representation of individuals with the most significant disease burden. While it is widely understood that RCTs are expensive to conduct, we suggest that if inclusion of racial minorities is a desired goal—which we believe it should be—then a greater effort needs to be made to ensure each racial group is adequately represented. A reasonable and just aim should be for representation of racial minorities in SLE RCTs that approximates the prevalence of SLE among these groups within the general population. Another option may be to conduct multiple studies with each one restricting to different race/ity groups. An argument can be made that a study design in which each group is equally represented, rather than proportional to disease prevalence, may be superior [33], though in reality, this may be difficult to achieve.

RCTs among SLE patients need to have larger sample sizes and the ability to conduct subgroup analyses to evaluate heterogeneity in outcomes among racial subgroups. In fact, our review found that smaller studies were less likely to include males and racial minorities, a dangerous notion as RCTs often inform clinical and treatment guidelines. It could be argued that the purpose of multiple small studies is to tightly control race and gender to remove the potential confounding; however, a favorable option could be conducting a series of small studies with targeted recruitment to circumvent this issue. We are also cognizant of the realities associated with SLE management including the varying organ involvement, heterogeneous disease manifestations, and the lack of consensus on clinically meaningful outcome measures. These realities present a unique set of challenges for the development of treatment guidelines. Nonetheless, it is imperative that novel strategies be developed to recruit racial minorities with SLE by identifying and improving barriers to enrollment into RCTs in order to better understand the disease’s diverse population.

## Appendix 1: Search Strategies

PubMed.

((((“Lupus Nephritis”[Mesh] OR “Lupus Erythematosus, Systemic”[Mesh])) OR lupus[tw])) AND (“Clinical Trial”[Publication Type] OR “Clinical Trials as Topic”[Mesh] OR clinical trial*[tiab])

Filters: Publication date from 1997/01/01 to 2017/07/31.

Cochrane Library of Systematic Reviews.

(TITLE (*lupus*) OR TITLE (*“Systemic lupus erythematosus”*) OR TITLE (*“Lupus nephritis”*)) AND (TITLE (*“clinical trial*”*))

Publication Year Between 1997 and 2017.

## References

[1] Mak A, Tay SH. Environmental factors, toxicants and systemic lupus erythematosus. Int J Mol Sci. [Internet]. Multidisciplinary Digital Publishing Institute (MDPI); 2014 [cited 2017 Nov 21];15:16043–56. Available from: http://www.ncbi.nlm.nih.gov/pubmed/25216337. 10.3390/ijms150916043 PMC4200809 25216337

[2] Weckerle CE, Niewold TB. The unexplained female predominance of systemic lupus erythematosus: clues from genetic and cytokine studies. Clin Rev Allergy Immunol. [Internet]. 2011 [cited 2017 Mar 27];40:42–9. Available from: http://link.springer.com/10.1007/s12016-009-8192-4 10.1007/s12016-009-8192-4 PMC2891868 20063186

[3] Murphy G, Isenberg D. Effect of gender on clinical presentation in systemic lupus erythematosus. Rheumatology (Oxford). [Internet]. Oxford University Press; 2013 [cited 2016 Aug 1];52:2108–15. Available from: http://www.ncbi.nlm.nih.gov/pubmed/23641038. 10.1093/rheumatology/ket160 23641038

[4] • Muñoz-Grajales C, González LA, Alarcón GS, Acosta-Reyes J. Gender differences in disease activity and clinical features in newly diagnosed systemic lupus erythematosus patients. Lupus [Internet]. 2016 [cited 2017 Nov 21];25:1217–23. Available from: http://www.ncbi.nlm.nih.gov/pubmed/26921269. **This study found that in newly diagnosed SLE patients, male gender is associated with higher disease activity despite the fact that male and female patients seem to experience similar overall disease manifestations.** 10.1177/0961203316635286 26921269

[5] Garcia MA, Marcos JC, Marcos AI, Pons-Estel BA, Wojdyla D, Arturi A, et al. Male systemic lupus erythematosus in a Latin-American inception cohort of 1214 patients. Lupus [Internet]. 2005 [cited 2017 Nov 21];14:938–46. Available from: http://www.ncbi.nlm.nih.gov/pubmed/16425573. 10.1191/0961203305lu2245oa 16425573

[6] Ding Y, He J, Guo J-P, Dai Y-J, Li C, Feng M, et al. Gender differences are associated with the clinical features of systemic lupus erythematosus. Chin Med J (Engl). [Internet]. 2012 [cited 2017 Nov 22];125:2477–81. Available from: http://www.ncbi.nlm.nih.gov/pubmed/22882925. 22882925

[7] Ward MM, Studenski S. Age associated clinical manifestations of systemic lupus erythematosus: a multivariate regression analysis. J Rheumatol. [Internet]. 1990 [cited 2017 Nov 24];17:476–81. Available from: http://www.ncbi.nlm.nih.gov/pubmed/2348426. 2348426

[8] •• Wise E, McCune J. Racial disparities result in unprecedented differences in outcomes for SLE patients. Rheumatol. [Internet]. 2015; Available from: http://www.the-rheumatologist.org/article/racial-disparities-in-outcome-for-sle-patients-are-unprecedented/. **This is a seminal paper examing the reasons why race still matters in lupus outcomes. The authors summarize key studies of disparties in the epidemiology and treatment of lupus.**

[9] Feldman CH, Hiraki LT, Liu J, Fischer MA, Solomon DH, Alarcón GS, et al. Epidemiology and sociodemographics of systemic lupus erythematosus and lupus nephritis among US adults with Medicaid coverage, 2000-2004. Arthritis Rheum. [Internet]. 2013 [cited 2016 Aug 1];65:753–63. Available from: http://www.ncbi.nlm.nih.gov/pubmed/23203603. 10.1002/art.37795 PMC3733212 23203603

[10] Dall’Era M, Cisternas MG, Snipes K, Herrinton LJ, Gordon C, Helmick CG. The Incidence and Prevalence of Systemic Lupus Erythematosus in San Francisco County, California: The California Lupus Surveillance Project. Arthritis Rheumatol. [Internet]. 2017 [cited 2017 Nov 22];69:1996–2005. Available from: http://www.ncbi.nlm.nih.gov/pubmed/28891237. 10.1002/art.40191 28891237

[11] •• Gómez-Puerta JA, Barbhaiya M, Guan H, Feldman CH, Alarcón GS, Costenbader KH. Racial/Ethnic variation in all-cause mortality among United States medicaid recipients with systemic lupus erythematosus: a Hispanic and asian paradox. Arthritis Rheumatol. (Hoboken, N.J.) [Internet]. 2015 [cited 2016 Aug 1];67:752–60. Available from: http://www.ncbi.nlm.nih.gov/pubmed/25590668. **This outstanding paper examined all-cause mortality by race/ethnicity among SLE patients in Medicaid. They characterized the Hispanic and Asian paradox - after accounting for demographic and clinical factors, Asian and Hispanic SLE Medicaid patients had lower mortality than did Blacks, Whites or Native American patients.** 10.1002/art.38981 PMC4366131 25590668

[12] Barbhaiya M, Feldman CH, Guan H, Gómez-Puerta JA, Fischer MA, Solomon DH, et al. Race/Ethnicity and Cardiovascular Events Among Patients With Systemic Lupus Erythematosus. Arthritis Rheumatol. [Internet]. 2017 [cited 2017 Dec 18];69:1823–31. Available from: http://www.ncbi.nlm.nih.gov/pubmed/28598016. 10.1002/art.40174 PMC6143392 28598016

[13] Hoppe C, Kerr D. Minority underrepresentation in cardiovascular outcome trials for type 2 diabetes. lancet. Diabetes Endocrinol. [Internet]. Elsevier; 2017 [cited 2017 Nov 24];5:13. Available from: http://www.ncbi.nlm.nih.gov/pubmed/28010783. 10.1016/S2213-8587(16)30324-2 28010783

[14] Oh SS, Galanter J, Thakur N, Pino-Yanes M, Barcelo NE, White MJ, et al. Diversity in Clinical and Biomedical Research: A Promise Yet to Be Fulfilled. PLOS Med. [Internet]. Public Library of Science; 2015 [cited 2017 Nov 24];12:e1001918. Available from: 10.1371/journal.pmed.1001918 10.1371/journal.pmed.1001918 PMC4679830 26671224

[15] Chen MS, Lara PN, Dang JHT, Paterniti DA, Kelly K. Twenty years post-NIH Revitalization Act: Enhancing minority participation in clinical trials (EMPaCT): Laying the groundwork for improving minority clinical trial accrual. Cancer [Internet]. 2014 [cited 2017 Nov 24];120:1091–6. Available from: http://www.ncbi.nlm.nih.gov/pubmed/24643646. 10.1002/cncr.28575 PMC3980490 24643646

[16] Zhang T, Tsang W, Wijeysundera HC, Ko DT. Reporting and representation of ethnic minorities in cardiovascular trials: A systematic review. Am Heart J. [Internet]. 2013 [cited 2017 Nov 22];166:52–7. Available from: http://www.ncbi.nlm.nih.gov/pubmed/23816021. 10.1016/j.ahj.2013.03.022 23816021

[17] Grant MJ, Booth A. A typology of reviews: an analysis of 14 review types and associated methodologies. Heal Inf Libr J. [Internet]. 2009 [cited 2018 Jan 16];26:91–108. Available from: http://www.ncbi.nlm.nih.gov/pubmed/19490148. 10.1111/j.1471-1842.2009.00848.x 19490148

[18] Census Bureau U. Race &amp; ethnicity what region of origin does census consider for each race category? 2017 [cited 2018 Jan 16]; Available from: https://www.census.gov/mso/www/training/pdf/race-ethnicity-onepager.pdf

[19] Izmirly PM, Wan I, Sahl S, Buyon JP, Belmont HM, Salmon JE, et al. The Incidence and Prevalence of Systemic Lupus Erythematosus in New York County (Manhattan), New York: The Manhattan Lupus Surveillance Program. Arthritis Rheumatol. [Internet]. 2017 [cited 2017 Nov 22];69:2006–17. Available from: http://doi.wiley.com/10.1002/art.40192 10.1002/art.40192 PMC11102806 28891252

[20] Somers EC, Marder W, Cagnoli P, Lewis EE, DeGuire P, Gordon C, et al. Population-based incidence and prevalence of systemic lupus erythematosus: the Michigan Lupus Epidemiology and Surveillance program. Arthritis Rheumatol. (Hoboken, N.J.) [Internet]. 2014 [cited 2016 Aug 2];66:369–78. Available from: http://www.ncbi.nlm.nih.gov/pubmed/24504809. 10.1002/art.38238 PMC4198147 24504809

[21] Zhou Y, Elashoff D, Kremen S, Teng E, Karlawish J, Grill JD. African Americans are less likely to enroll in preclinical Alzheimer’s disease clinical trials. Alzheimer’s Dement. Transl Res Clin Interv. [Internet]. Elsevier; 2017 [cited 2018 Jan 17];3:57–64. Available from: https://www.sciencedirect.com/science/article/pii/S2352873716300397. 10.1016/j.trci.2016.09.004 PMC5651355 29067319

[22] Sturgeon KM, Hackley R, Fornash A, Dean LT, Laudermilk M, Brown JC, et al. Strategic recruitment of an ethnically diverse cohort of overweight survivors of breast cancer with lymphedema. Cancer [Internet]. 2018 [cited 2018 Jan 17];124:95–104. Available from: http://www.ncbi.nlm.nih.gov/pubmed/28881471. 10.1002/cncr.30935 PMC5743016 28881471

[23] Napoles A, Cook E, Ginossar T, Knight KD, Ford ME. Applying a Conceptual Framework to Maximize the Participation of Diverse Populations in Cancer Clinical Trials. Adv Cancer Res. [Internet]. Academic Press; 2017 [cited 2018 Jan 17];133:77–94. Available from: https://www.sciencedirect.com/science/article/pii/S0065230X16300719. 10.1016/bs.acr.2016.08.004 PMC5542779 28052822

[24] Niranjan SJ, Durant RW, Wenzel JA, Cook ED, Fouad MN, Vickers SM, et al. Training Needs of Clinical and Research Professionals to Optimize Minority Recruitment and Retention in Cancer Clinical Trials. J Cancer Educ. [Internet]. 2017 [cited 2018 Jan 17]; Available from: http://www.ncbi.nlm.nih.gov/pubmed/28776305. 10.1007/s13187-017-1261-0 PMC5797508 28776305

[25] Paasche-Orlow MK, Wolf MS. Promoting Health Literacy Research to Reduce Health Disparities. J Health Commun. [Internet]. Taylor & Francis Group; 2010 [cited 2017 Nov 22];15:34–41. Available from: http://www.tandfonline.com/doi/abs/10.1080/10810730.2010.499994. 10.1080/10810730.2010.499994 20845191

[26] González L, Toloza S, McGwin G, Alarcón G. Ethnicity in systemic lupus erythematosus (SLE): its influence on susceptibility and outcomes. Lupus [Internet]. SAGE PublicationsSage UK: London, England; 2013 [cited 2017 Nov 22];22:1214–24. Available from: http://journals.sagepub.com/doi/10.1177/0961203313502571. 10.1177/0961203313502571 24097993

[27] Kelly JA, Kelley JM, Kaufman KM, Kilpatrick J, Bruner GR, Merrill JT, et al. Interferon regulatory factor-5 is genetically associated with systemic lupus erythematosus in African Americans. Genes Immun. [Internet]. 2008 [cited 2017 Nov 22];9:187–94. Available from: http://www.ncbi.nlm.nih.gov/pubmed/18288123. 10.1038/gene.2008.4 18288123

[28] Lee YH, Rho YH, Choi SJ, Ji JD, Song GG. The functional p53 codon 72 polymorphism is associated with systemic lupus erythematosus. Lupus [Internet]. 2005 [cited 2017 Nov 22];14:842–5. Available from: http://www.ncbi.nlm.nih.gov/pubmed/16302680. 10.1191/0961203305lu2224oa 16302680

[29] Lee YH, Harley JB, Nath SK. CTLA-4 polymorphisms and systemic lupus erythematosus (SLE): a meta-analysis. Hum. Genet. [Internet]. 2005 [cited 2017 Nov 22];116:361–7. Available from: http://www.ncbi.nlm.nih.gov/pubmed/15688186. 10.1007/s00439-004-1244-1 15688186

[30] Ortega VE, Meyers DA. Pharmacogenetics: Implications of race and ethnicity on defining genetic profiles for personalized medicine. J Allergy Clin Immunol. [Internet]. 2014 [cited 2017 Nov 24];133:16–26. Available from: http://www.ncbi.nlm.nih.gov/pubmed/24369795. 10.1016/j.jaci.2013.10.040 PMC3933289 24369795

[31] Gage BF, Bass AR, Lin H, Woller SC, Stevens SM, Al-Hammadi N, et al. Effect of Genotype-Guided Warfarin Dosing on Clinical Events and Anticoagulation Control Among Patients Undergoing Hip or Knee Arthroplasty. JAMA [Internet]. American Medical Association; 2017 [cited 2017 Nov 23];318:1115. Available from: http://jama.jamanetwork.com/article.aspx?doi=10.1001/jama.2017.11469 10.1001/jama.2017.11469 PMC5818817 28973620

[32] •• Eggener S. Generalizability of clinical trials: why it matters for patients and public policy. Eur Urol. [Internet]. Elsevier; 2017 [cited 2017 Nov 23];71:515–6. Available from: http://www.ncbi.nlm.nih.gov/pubmed/27743755. **This paper succintly explains the importance of external validity of RCTs.** 10.1016/j.eururo.2016.09.049 27743755

[33] Rothman KJ, Gallacher JE, Hatch EE. Why representativeness should be avoided. Int. J. Epidemiol. [Internet]. 2013 [cited 2017 Nov 23];42:1012–4. Available from: http://www.ncbi.nlm.nih.gov/pubmed/24062287. 10.1093/ije/dys223 PMC3888189 24062287

